# Improving mental health outcomes through online brain health training in adults with or without mental illness

**DOI:** 10.3389/fpsyg.2026.1826717

**Published:** 2026-06-04

**Authors:** Sarah A. Laane, Lori G. Cook, Jeffrey S. Spence, Sandra Bond Chapman

**Affiliations:** Center for BrainHealth^®^, School of Behavioral and Brain Sciences, The University of Texas at Dallas, Dallas, TX, United States

**Keywords:** brain health, cognitive training, digital health, mental health promotion, prevention

## Abstract

**Background:**

Mental health promotion aims to improve well-being and quality of life for individuals and communities. However, most existing interventions target specific populations and are designed to address particular symptoms or diagnoses, limiting their reach to the general population. Therefore, there is a need to better understand the potential benefits of universal mental health promotion programs.

**Objective:**

This quasi-experimental pre-post study with matching examined the impact of an online brain health training as a mental health promotion tool for adults with and without mental illness. The program included Strategic Memory Advanced Reasoning Training (SMART), an executive function strategy training, combined with education on daily practices that support overall health.

**Methods:**

Participants included a matched sample of 370 adults (185 with and 185 without mental illness), ages 18–87. Linear mixed-effects models were used to (1) examine baseline group differences in mental health outcomes (psychological distress, resilience, quality of life, and engagement in meaningful activities) and high-level cognitive function, operationalized as a composite measure of complex cognition (“clarity”); (2) evaluate changes in these outcomes following 6 months of online brain health training; and (3) assess associations between changes in cognitive clarity and mental health outcomes over time.

**Results:**

At baseline, participants with mental illness reported greater psychological distress, lower resilience, poorer quality of life, reduced engagement in meaningful activities, and lower cognitive clarity compared with those without mental illness. Following training, both groups demonstrated significant improvements, including reduced psychological distress and increased resilience, quality of life, and engagement in meaningful activities. Clarity improved only among participants without mental illness who completed at least the core SMART training. Across the full sample and within both groups, improvements in clarity were significantly associated with improvements in mental health outcomes, with some group differences observed.

**Conclusion:**

Online brain health training warrants further study as a mental health promotion intervention, with potential to complement standard care for individuals with mental illness and prevent emerging concerns among those without a diagnosis. Delivering this training online offers a promising, accessible pathway for population-level mental health promotion.

## 1 Introduction

We are in the midst of an escalating mental health crisis. The prevalence of mental disorders has risen steadily worldwide, with global cases nearly doubling over the past three decades (Fan et al., 2025). In the United States, more than one in five adults live with a mental health condition [National Institute of Mental Health (NIMH), 2023], and approximately 20% of the general population report symptoms of anxiety or depression (Terlizzi and Zablotsky, 2024). Individuals with pre-existing mental health conditions report worsening symptoms, while psychological well-being in the general population continues to decline (Reinert et al., 2021; Substance Abuse and Mental Health Services Administration, 2020; Vindegaard and Benros, 2020). One approach to alleviating this growing crisis is through public mental health promotion. Mental health promotion seeks to strengthen individual competencies and psychological resources while enhancing community assets to improve well-being and quality of life, and reduce risk for mental disorder (Kobau et al., 2011). Grounded in positive psychology, this approach shifts focus from symptom reduction alone to the cultivation of strengths, resilience, and supportive environments that enable individuals and communities to thrive (Barry, 2019; Kobau et al., 2011).

### 1.1 Mental health promotion

Mental health promotion is increasingly recognized as a core component of the mental health care continuum, serving both as a preventive strategy for individuals without mental illness and as a complementary approach alongside clinical treatment (Barry, 2019; Substance Abuse and Mental Health Services Administration, n.d.). Unlike prevention and treatment models, which primarily target symptom identification and reduction, promotion strategies aim to strengthen well-being regardless of diagnostic status. In this way, mental health promotion can be both a proactive approach to reducing mental distress and a supportive approach to bolstering mental health regardless of one’s current diagnostic status. Importantly, promotion is not positioned as a replacement for clinical treatment but as a complementary tool that can optimize outcomes when integrated alongside traditional care (Jané-Llopis et al., 2005; Kobau et al., 2011; Singh et al., 2022). Mental health promotion interventions include a wide range of lifestyle and behavioral strategies including but not limited to those related to exercise (Gordon et al., 2018), nutrition (Suárez-López et al., 2023), sleep quality (Gee et al., 2019), social connection or peer support programs (Smit et al., 2023), mental health literacy programming (Reis et al., 2022), gratitude practices (Watkins et al., 2014) and psychological therapeutic interventions such as community programs based in cognitive-behavioral therapy (Nakagami et al., 2024).

Despite this breadth, there remains a critical need for evidence-based, accessible, and low-cost mental health promotion interventions suitable for the general population (Jané-Llopis et al., 2005; Kobau et al., 2011; Singh et al., 2022). Many existing programs target specific conditions or populations, such as individuals with chronic illness (Thompson et al., 2010), college students (Zhan, 2025), or postpartum mothers (Elliott et al., 2000), limiting their reach. Developing scalable promotion interventions that can benefit individuals across mental health statuses is therefore essential for advancing population-level mental health.

### 1.2 SMART brain health training for mental health promotion

Strategic Memory Advanced Reasoning Training (SMART) is a strategy-based executive function training program that was originally designed to improve cognitive performance by targeting higher-order cognitive control processes mediated by frontal lobe networks, with evidence of cognitive gains across various clinical and non-clinical populations (Anand et al., 2010; Chapman et al., 2021; Gamino et al., 2009; Vas et al., 2016; Young et al., 2021; Venza et al., 2016). However, evidence also suggests SMART may also bolster mental health (Laane et al., 2022; Vas et al., 2016; Young et al., 2021). This was first observed in studies of in-person SMART interventions (Vas et al., 2016; Young et al., 2021), however more recently SMART was transformed to be delivered online, making it ideal as a low-cost and highly accessible mental health promotion intervention (Chapman et al., 2021). A previous examination of online SMART’s impact as a mental health promotion intervention found that healthy adults during the Covid-19 pandemic who engaged in twelve weeks of online SMART had a significant reduction in depression, anxiety, and stress symptoms (Laane et al., 2022). There is a need to extend this work to determine if SMART could be a promotion tool that is effective for the broader population, including those with pre-existing mental illness.

SMART may be an especially impactful mental health promotion intervention because not only does it show potential to decrease symptoms of psychological distress, but SMART also enhances cognitive function. Cognitive deficits have been observed transdiagnostically across individuals with mental illnesses (Hilton et al., 2024; Keefe et al., 2011; Keshavan et al., 2014; Perini et al., 2019; Qureshi et al., 2011; Rock et al., 2014; Vinogradov et al., 2012; Zainal and Newman, 2018). In some cases, cognitive deficits are one of the first symptoms of mental illness to be observed. For example, cognitive impairment serves as a predictor for the onset of psychosis (Karcher et al., 2023; Reichenberg, 2005). Additionally, cognitive training (e.g., cognitive remediation, cognitive rehabilitation) interventions have been shown to improve well-being for those with mental illness, including those with schizophrenia (McGurk et al., 2007; Medalia et al., 1998, 2000; Twamley et al., 2003; Vauth et al., 2005), major depressive disorder (Browning et al., 2011; Motter et al., 2016; Woolf et al., 2022), and anxiety disorder (Beard et al., 2012). Therefore, by deploying a promotion intervention that is designed to support both mental well-being and cognitive function, it may be possible to enhance both features of mental illness with the same intervention.

### 1.3 The present study

The current study aimed to expand upon previous work by investigating the impact of an online SMART brain health training program on mental health and cognitive function in adults with and without self-reported diagnosed mental illness. Mental health was measured on four dimensions: symptoms of psychological distress, resilience, quality of life, and engagement in meaningful activities. Cognitive function was measured using a composite score of high-level cognitive function (clarity) from the multidimensional Brain Health Index measure (Chapman et al., 2021; Cook et al., 2025). The research was motivated by the growing demand for scalable mental health promotion interventions, particularly those appropriate for broader community use.

The first aim was to examine baseline differences in mental health and cognitive performance between individuals with and without mental illness. It was hypothesized that those with self-reported mental illness would exhibit greater symptoms of psychological distress and lower levels of resilience, quality of life, engagement in meaningful activities, and clarity at baseline. Understanding these initial vulnerabilities is essential for developing tailored mental health promotion approaches to address capacity building in both groups. The second aim was to measure changes in mental health and cognitive function following the online brain health training in both groups. It is expected that all participants will show improvement in both mental health and cognitive function, but that the magnitude of improvement will be greater for those with mental illness, given the likelihood that this group with have lower baseline functioning. Additionally, this aim explored the influence of age, gender, education, and training utilization on changes in mental health and cognitive performance following the training to inform our understanding as to whether the training would be appropriate for widespread public mental health promotion or more effective when targeted to specific populations. The third aim examined whether improvements in cognitive function (clarity) predict improvements in mental health outcomes. This analysis enhanced understanding of the relationship between mental health and cognitive function. It was hypothesized that there would be a positive correlation between gains in clarity and gains in symptoms of psychological distress, resilience, quality of life, and engagement in meaningful activities for those with and without mental illness. Together, these aims advance our understanding of the potential of brain health training as a universal mental health promotion intervention.

## 2 Methods

### 2.1 Design

This study employed a two-group, non-randomized, quasi-experimental pre-post test design using data from the BrainHealth Project (Chapman et al., 2021; Cook et al., 2025), a longitudinal research initiative launched by the Center for BrainHealth at The University of Texas at Dallas. The BrainHealth Project aims to evaluate and refine indices of brain health, assess the impact of evidence-based interventions, and explore mechanisms of brain health across the adult lifespan. Participants complete comprehensive online assessments and engage in brain health training programs, including a 6-month online Strategic Memory Advanced Reasoning Training (SMART) program, which provided the context to explore this study’s aims. This study was conducted in accordance with the guidelines of the Institutional Review Board of the University of Texas at Dallas.

To reduce the impact of confounding variables, a quasi-experimental design was implemented using propensity score matching. Participants were matched on gender, age (grouped into quartiles), and education level using one-to-one nearest neighbor matching (Rosenbaum and Rubin, 1983; Ho et al., 2011). This approach allowed for comparison of demographically similar individuals across mental illness status.

### 2.2 Participants

**Figure 1:**
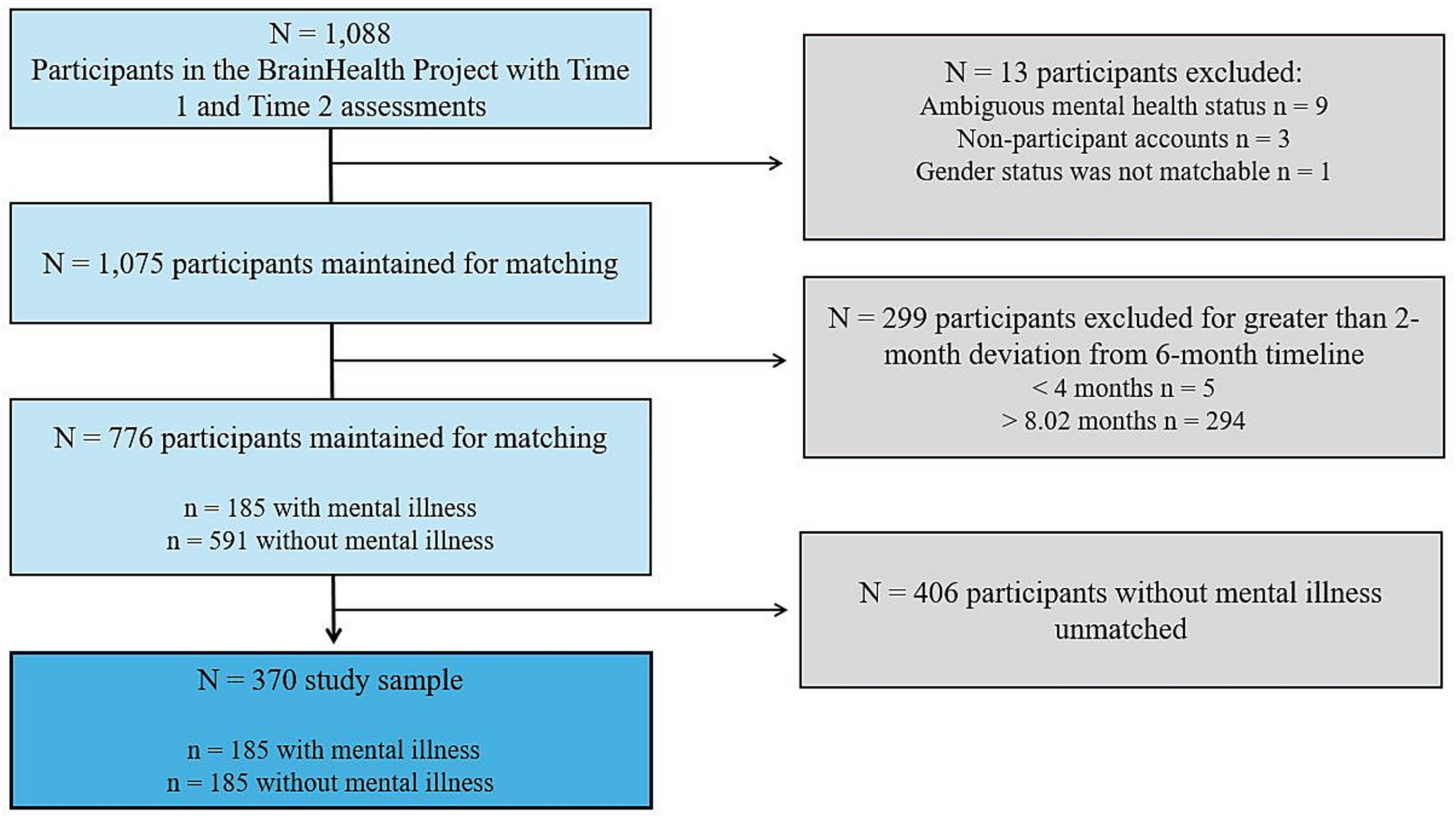
Participant selection.

The initial sample included 1,088 adults (ages 18–87) who completed both pre- and post-training assessments (see Figure 1). General inclusion criteria for the BrainHealth Project required participants to be English-speaking, have internet access, and be able to interact with the online platform. Exclusion criteria included diagnosis of a neurodegenerative disease, history of stroke or brain injury impairing current function, or autism spectrum disorder that affects individual functioning.

For this study, additional inclusion criteria required completion of both assessments within two months of the 6-month training period. Participants were excluded if they had atypical training protocols, ambiguous mental illness status, or duplicate/non-participant accounts. One participant was excluded due to a unique gender response that precluded matching. After exclusions, 776 participants remained: 185 with self-reported mental illness and 591 without. Propensity score matching yielded a final sample of 370 participants (185 per group).

Participants reported their mental health conditions by responding to the question, “Do you currently have any of the following diagnoses?” and selecting from a list of mental illnesses. Options included “none” and “other,” the latter with a text box for unlisted diagnoses. Individuals self-reporting clinical depression, addiction, post-traumatic stress disorder (PTSD), anxiety or panic disorder, eating disorder, obsessive-compulsive disorder (OCD), bipolar disorder, schizophrenia, or another qualifying mental illness were classified within the mental illness group. Participants who selected “none” or whose “other” responses were not mental illnesses (per DSM-5 TR; American Psychiatric Association, 2022) were classified within the without mental illness group. Diagnoses of learning disability, attention-deficit/hyperactivity disorder (ADHD), or autism spectrum disorder alone did not qualify for the mental illness group, as the study focused on psychiatric illnesses rather than neurodiversity or developmental conditions.

### 2.3 Propensity score matching

Propensity score matching was conducted using the MatchIt package in R (Ho et al., 2011; R Core Team, 2020; RStudio Team, 2022), following guidelines from Randolph et al. (2014). Participants were matched on age, gender, and education level using one-to-one nearest neighbor matching. The matching procedure effectively reduced mean differences across covariates to below 0.03, indicating good balance between groups (see Supplementary Figure S1). Visual diagnostics, including jitter plots and histograms, confirmed successful matching, demonstrating similar propensity score distributions between groups (see Supplementary Figure S2).

### 2.4 Procedure

Participants were recruited into the BrainHealth Project through word of mouth, the study website, email, and social media. This study focused on their first six months of participation, during which they completed online assessments, SMART cognitive training, and virtual coaching via the BrainHealth Project platform.

Following the online consent process, as approved by The University of Texas at Dallas’ Institutional Review Board, participants completed the BrainHealth Index (BHI), a multidimensional online assessment of cognitive performance and self-reported well-being. The BHI takes approximately 60–90 min and can be completed over multiple sittings within a two-week window. After completing the BHI, participants were offered the opportunity to complete a 20-min virtual coaching session with a trained clinician to review their BHI results, orient them to the platform, and introduce the SMART training.

Strategic Memory Advanced Reasoning Training (SMART) is a self-paced, top-down executive function training. The training is composed of five core modules which includes 4–5 h of content across 29 lessons (see Table 1) that teach strategic attention, integrated reasoning, and innovation through short videos and interactive exercises (Chapman et al., 2021; Cook et al., 2025). Completion of these five modules was defined as “core SMART.” Participants were encouraged to engage daily for 5–15 min.

**Table 1:** Overview of the core SMART training curriculum.

Sections	Lessons	Training content
Introduction	1	Introduction
Strategic attention	2	Introduction to strategic attention
	3–4	Brainpower of two
	5–7	Brainpower of one
	8–10	Brainpower of none
	11	Summary
Integrated reasoning	12	Introduction to integrated reasoning
	13–15	Integrated reasoning strategies
	16	Summary
Innovation	17	Introduction to innovation
	18–19	Brainpower of infinite
	20–22	Brainpower of paradox
	23–25	Brainpower of unknown
	26	Innovation benefits
Application of SMART strategies	27–28	Brain fitness formula
	29	Strategies recap

Core Strategic Memory Advanced Reasoning Training (SMART) modules represent approximately 4–5 h of online strategy-based executive function training.

After core SMART, participants could access additional modules linking SMART strategies to stress management, sleep hygiene, and daily life applications. Participants also had access to daily healthy habit tracking and curated educational resources (e.g., TED Talks, research articles) to support integration of strategies into everyday life. A midpoint coaching session was offered to reinforce strategy use and help participants set personal goals. Six months after the initial BHI, participants completed a second BHI to assess changes in cognitive and mental health outcomes. The BHI has demonstrated suitability for test–retest designs, with evidence indicating minimal susceptibility to practice effects. Specifically, participants in waitlist control conditions and those with limited training exposure do not exhibit significant score improvements (Young et al., 2025; Chapman et al., 2021).

### 2.5 Mental health measures

#### 2.5.1 Symptoms of psychological distress

Symptoms of psychological distress were assessed using the *Depression Anxiety Stress Scale-21* (DASS-21; Lovibond and Lovibond, 1995; Henry and Crawford, 2005), a self-report tool measuring depression, anxiety, and stress symptoms over a one-week period. The 21-item scale includes seven items per symptom type, rated on a 4-point Likert scale (0 = Never to 3 = Almost Always). The DASS-21 has demonstrated strong reliability and construct validity across both clinical and non-clinical populations for capturing symptoms of psychological distress (Antony et al., 1998; Henry and Crawford, 2005; Ng et al., 2007).

#### 2.5.2 Resilience

Resilience was assessed using the *Connor-Davidson Resilience Scale* (CD-RISC-25; Connor and Davidson, 2003), a 25-item self-report measure of stress-coping ability over the past month. Items are rated on a 5-point Likert scale (0–4), with higher scores indicating greater resilience. The CD-RISC-25 has shown strong reliability and validity in both general and clinical populations (Connor and Davidson, 2003).

#### 2.5.3 Quality of life

Quality of life was measured using the *Quality of Life Scale* (QOLS; Burckhardt and Anderson, 2003), a 16-item self-report tool assessing satisfaction across six domains: physical well-being, relationships, social and civic activities, personal development, recreation, and independence. Items are rated on a 7-point scale (1 = terrible to 7 = delighted), with higher scores indicating better quality of life. The QOLS has shown good reliability and construct validity in both clinical and community samples (Burckhardt and Anderson, 2003).

#### 2.5.4 Engagement in meaningful activities

Engagement in meaningful activities was measured using the *Engagement in Meaningful Activities Survey* (EMAS; Goldberg et al., 2002; Eakman, 2011), a 12-item self-report measure of current participation in meaningful activities. Items begin with “The activities I do…” followed by a statement of meaningfulness (e.g., “…reflect the kind of person I am”). Responses are rated on a 4-point Likert scale (1 = Rarely to 4 = Always), with higher scores indicating greater engagement. The EMAS has shown good reliability and validity in community samples including individuals with mental illness (Eakman et al., 2010; Eakman, 2011; Goldberg et al., 2002).

### 2.6 Cognitive performance measure

#### 2.6.1 Clarity

Clarity is the *BrainHealth Index* composite score reflecting high-level functional cognitive performance (Chapman et al., 2021, 2022). It represents an individual’s *readiness to reason* by demonstrating the ability to apply metacognitive strategies to reason through complex problems, create new solutions/see opportunities and move beyond rote (habitual) processing to use critical thinking. Clarity integrates data from real-time cognitive performance tasks and self-reported real-life cognitive functioning and sleep quality. Given sleep’s significant influence on cognition, self-reported sleep quality is included as a foundational component. See Chapman et al. (2021) and Cook et al. (2025) for additional information on clarity components.

### 2.7 Overview of analytic analyses

Statistical analyses were conducted using R (R Core Team, 2020) and RStudio (v.2022.7.01; RStudio Team, 2022), utilizing packages lsr (Navarro, 2015), nlme (Pinheiro et al., 2023) and Tidyverse (Wickham et al., 2019). Tidyverse’s ggplot2 (Wickham, 2016), patchwork (Pedersen, 2025) and cowplot (Wilke, 2025) used for images. For aims one and two, a linear mixed-effects model was used to assess main effects of time (pre- vs. post-SMART intervention) and the interactions between age (centered), gender, education (less than bachelor’s, bachelor’s, greater than bachelor’s), training utilization (log-transformed, centered), and mental illness status (with vs. without mental illness) on mental health outcomes (psychological distress, resilience, quality of life, and meaningful activity engagement), with participants modeled as random effects. Interactions were modeled between mental illness status and training utilization, and between time and mental illness status, age, gender, and education. To assess aim three, linear regressions were conducted to test whether changes in clarity from pre- to post-intervention predicted changes in mental health outcomes over the same period. We accounted for multiple testing by controlling the false discovery rate (FDR) using the adaptive linear step-up procedure proposed by Benjamini et al. (2006).

## 3 Results

### 3.1 Descriptive statistics

After matching, the sample comprised 185 participants with mental illness and 185 without. In the mental illness group, clinical depression and anxiety disorder were most common (Table 2). Comorbidities were present: 71% had one diagnosis (*n* = 131), 17.84% had two (*n* = 33), and 11.35% had three or more diagnosis. Gender, age, and education were balanced across groups. Training utilization differed significantly, with those without mental illness completing more sections (M = 11.19) than those with mental illness (M = 8.49). Although Online SMART encourages daily use, its self-paced format produced wide variation (0–94 sections completed). However, about half of each group completed the core SMART curriculum, 51% without mental illness (*n* = 95) and 44% with mental illness (*n* = 82), indicating similar engagement with the foundational cognitive strategies.

**Table 2:** Participant demographics by mental illness status.

Demographics	Without mental illness	With mental illness	*p*-value
	(*n* = 185)	(*n* = 185)	
Gender			
Female	150 (81.08%)	152 (82.16%)	0.893
Male	35 (18.92%)	33 (17.84%)	
Age			
Mean	62.31	61.26	0.452
SD	13	13.82	
Range	25.1–86.09	18.07–87.31	
Education			
Less than bachelor’s	39 (21.08%)	38 (20.54%)	0.991
Bachelor’s degree	53 (28.65%)	53 (28.65%)	
Greater than bachelor’s	93 (50.27%)	94 (50.81%)	
Training utilization (in sections)			
Mean	11.19	8.49	0.036*
SD	13.84	10.65	
Range	0–94	0–50	
Race			
Asian	9 (4.86%)	1 (0.54%)	0.132
Black or African American	3 (1.62%)	5 (2.70%)	
Mixed race	7 (3.78%)	7 (3.78%)	
Other/Unknown	5 (2.70%)	6 (3.24%)	
White	161 (87.03%)	166 (89.73%)	
Mental illness diagnoses**			
Addiction	-	14 (7.57%)	
Anxiety/panic disorder	-	80 (43.24%)	
Bipolar disorder	-	12 (6.49%)	
Clinical depression	-	104 (56.22%)	
Eating disorder	-	5 (2.70%)	
OCD	-	16 (8.65%)	
PTSD	-	39 (21.08%)	
Schizophrenia/Schizoaffective disorder	-	1 (0.54%)	
Other	-	2 (1.08%)	

SD: Standard deviation. *Significant difference at *p* < 0.05. **Participants may report multiple disorders and are thus counted in multiple categories. OCD: Obsessive-compulsive disorder. PTSD: Post-traumatic stress disorder.

### 3.2 Aim 1: baseline mental health and clarity

**Figure 2:**
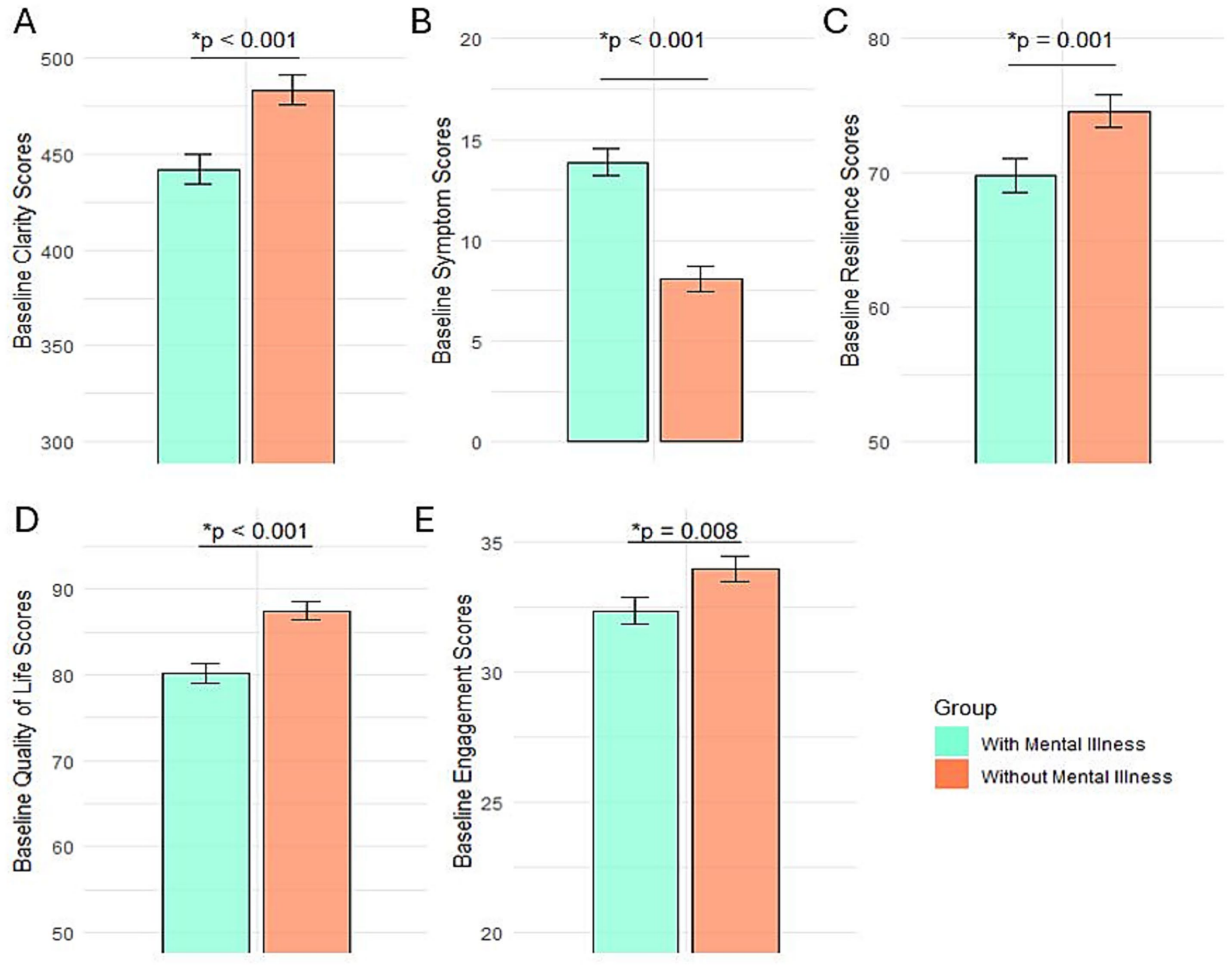
Baseline mean differences in clarity and mental health outcomes by mental illness status. For panels **(A,C,D,E)**, higher scores represent more favorable outcomes. For panel **(B)**, higher scores represent more symptoms of psychological distress and therefore indicate a less favorable outcome.

At baseline, participants with mental illness reported significantly poorer outcomes across all measures compared to those without mental illness. They had higher psychological distress (*M* = 13.87 vs. 8.09; *t*(362) = 7.33, *p* < 0.001, *d* = 0.77), lower resilience (*M* = 69.82 vs. 74.61; *t*(362) = −3.22, *p* = 0.001, *d* = 0.34), lower quality of life (*M* = 80.18 vs. 87.49; *t*(362) = −5.46, *p* < 0.001, *d* = 0.57), and less engagement in meaningful activities (*M* = 32.37 vs. 33.99; *t*(362) = −2.67, *p* = 0.008, *d* = 0.29). They also scored lower in clarity (*M* = 442.07 vs. 483.28; *t*(362) = −4.48, *p* < 0.001, *d* = 0.47) (see Figure 2).

### 3.3 Aim 2: change in mental health and clarity

**Figure 3:**
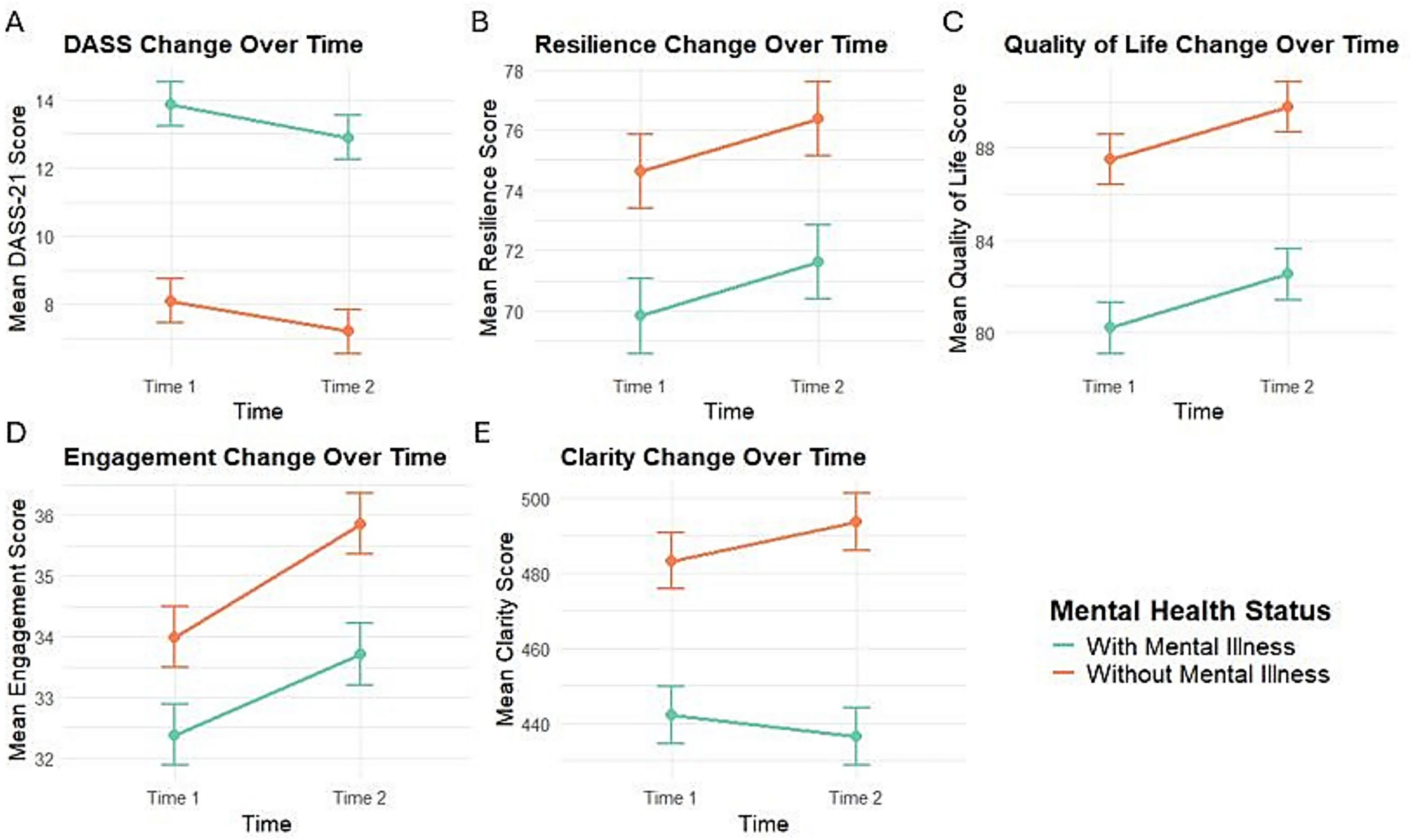
Changes in mental health outcomes and clarity by mental illness status from pre to post brain health training. For panel **(A)**, higher scores represent greater symptoms of psychological distress and therefore indicate a less favorable outcome. For panels **(B–E)**, higher scores represent more favorable outcomes.

Figure 3 illustrates changes in clarity and mental health outcomes by mental illness status from pre- to post-brain health training. All mental health measures improved significantly regardless of mental health status. Specifically, symptoms of psychological distress, resilience, quality of life, and engagement in meaningful activities improved significantly from baseline (Time 1) to post-test (Time 2). See Table 3 for group means and Table 4 for time main effect. Improvements in mental health outcomes were comparable for participants with and without a mental illness diagnosis, with no significant Time × Mental Illness Interaction effects (see Table 4).

**Table 3:** Changes in mental health outcomes and clarity from baseline to post-test by mental illness status.

Mental health outcome	Mental illness status	Time 1 Mean (SE)	Time 2 Mean (SE)	Cohen’s *d*	95% CI for Cohen’s *d*
Symptoms of psychological distress	Without MI	8.09 (0.65)	7.20 (0.65)	−0.21	(−0.41, −0.00)
	With MI	13.87 (0.66)	12.89 (0.66)	−0.23	(−0.43, −0.02)
Resilience	Without MI	74.61 (1.23)	76.37 (1.23)	0.27	(0.06, 0.47)
	With MI	69.82 (1.24)	71.62 (1.24)	0.27	(0.07, 0.48)
Quality of life	Without MI	87.49 (1.10)	89.78 (1.10)	0.36	(0.15, 0.57)
	With MI	80.18 (1.12)	82.51 (1.12)	0.36	(0.15, 0.57)
Engagement in meaningful activities	Without MI	33.99 (0.50)	35.85 (0.50)	0.57	(0.36, 0.78)
	With MI	32.37 (0.50)	33.71 (0.50)	0.40	(0.20, 0.61)
Clarity	Without MI	483.28 (7.59)	493.50 (7.59)	0.18	(−0.03, 0.38)
	With MI	442.07 (7.66)	436.32 (7.66)	−0.10	(−0.30, 0.11)

Cohen’s *d* represents within-group change from baseline to post-test. MI = mental illness diagnosis. CI = confidence interval.

**Table 4:** Linear mixed-effects full model for mental health outcomes and clarity.

Predictor	Psychological distress	Resilience	Quality of life	Engagement in meaning activities	Clarity
Time	−0.94 (0.42), −2.23(364), 0.026*	1.78 (0.64), 2.77(364), 0.006**	2.31 (0.63), 3.69(364), <0.001**	1.60 (0.32), 4.97(364), <0.001**	2.24 (5.67), 0.39(364), 0.694
Time × Mental illness interaction	−0.08 (0.63), −0.13(364), 0.894	0.04 (0.97), 0.04(364), 0.966	0.04 (0.94), 0.04(364), 0.966	−0.53 (0.48), −1.10(364), 0.273	−15.96 (8.55) −1.87(364) 0.063~
Training	−0.59 (0.28), −2.10(362), 0.037*	0.94 (0.55), 1.72(362), 0.087	1.32 (0.49), 2.71(362), 0.007**	0.42 (0.22), 1.94(362), 0.053~	10.08 (3.17) 3.18(362) 0.002**
Mental illness × Training interaction	−0.56 (0.56), −1.00(362), 0.320	−0.03 (1.09), −0.03(362), 0.979	1.40 (0.97), 1.44(362), 0.152	0.35 (0.43), 0.82(362), 0.414	1.95 (6.33) 0.31(362) 0.758

Values are presented as estimate (standard error), t-value (df), and *p*-value. **Statistically significant based on control of the false discovery rate (FDR) = 3%; * FDR = 9%; ~ FDR = 11%.

A higher degree of training utilization was associated with greater gains across measures. Significant effects were observed for all outcomes except resilience, which showed a marginal association (*p* = 0.087; see Table 4, training). Demographic variables showed minimal effects (see Supplementary Table S1). Being of a younger age was associated with greater gains in resilience and clarity, while education moderated resilience gains only. Gender did not significantly moderate change in any outcome.

In contrast to the mental health outcomes, clarity did not change significantly from baseline to post-test in the overall sample (see Table 4, time). This is explained by the interaction effect (see Table 4, time × mental illness interaction for clarity) where clarity improved only among participants without mental illness, and not for those with mental illness. See mean changes in Table 3. Additionally, greater training utilization was associated with higher clarity (see Table 4, training) regardless of group (see Table 4, mental illness × training interaction).

To further examine whether training dosage influenced clarity outcomes, analyses were repeated in the subsample of participants who completed at least the five core SMART training sections (*n* = 177; 82 with mental illness, 95 without). In this model, clarity change for the full subsample remained non-significant, *p* = 0.240, however participants without mental illness demonstrated an increase in clarity that was marginally significant at *p* = 0.067. Mean clarity scores increased for both groups (see Supplementary Table S2). The effect size for participants without mental illness was small (Cohen’s *d* = 0.23), suggesting that a larger dose of SMART cognitive training may be required to produce meaningful improvement in clarity.

### 3.4 Aim 3: does change in clarity predict change in mental health

For the overall sample, clarity change significantly predicted reductions in symptoms of psychological distress (*p* < 0.001, R^2^ = 0.032), increases in resilience (*p* < 0.001, R^2^ = 0.053), quality of life (*p* < 0.001, R^2^ = 0.036), and engagement in meaningful activities (*p* = 0.002, R^2^ = 0.023). See Table 5. While significant, these effects explained only a small portion of the variance, indicating that other factors also contributed to changes in mental health outcomes. See Supplementary Figure S3 for visualization of findings.

**Table 5:** Multiple linear regressions predicting change in mental health outcomes from change in clarity.

Outcome	Sample	b	SE	t	p*	R^2^
Symptoms of psychological distress	Overall	0.01	0.004	3.51	<0.001	0.032
	With MI	0.02	0.007	3.14	0.002	0.051
	Without MI	0.01	0.001	1.72	0.088	0.016
Resilience	Overall	0.03	0.006	4.53	<0.001	0.053
	With MI	0.03	0.009	3.34	0.001	0.058
	Without MI	0.02	0.008	3.11	0.002	0.050
Quality of life	Overall	0.02	0.006	3.71	<0.001	0.036
	With MI	0.02	0.009	2.50	0.013	0.033
	Without MI	0.02	0.007	2.81	0.005	0.041
Meaningful activity engagement	Overall	0.01	0.003	3.08	0.002	0.023
	With MI	0.01	0.004	1.72	0.087	0.016
	Without MI	0.01	0.004	2.66	0.009	0.037

Positive *b* values indicate that increases in clarity were associated with greater improvements in the outcome. Change in symptoms of psychological distress was calculated as time 1 DASS-21 results minus time 2 DASS-21 results, such that higher values indicated more reduction in symptoms. MI = mental illness. *FDR = 2% across all *p*-values.

When analyzed by mental illness status, clarity change remained a significant predictor of resilience and quality of life for both groups. For those with mental illness, clarity change also predicted reductions in psychological distress (*p* = 0.002, R^2^ = 0.051) but did not predict engagement in meaningful activities. Conversely, for those without mental illness, clarity change predicted improvements in resilience (*p* = 0.002, R^2^ = 0.050), quality of life (*p* = 0.005, R^2^ = 0.041), and engagement in meaningful activities (*p* = 0.009, R^2^ = 0.037) but not reductions in psychological distress. Overall, these results support the hypothesis that increased clarity is associated with improvements in key mental health components, although the predictive effects are modest and vary depending on mental illness status.

## 4 Discussion

The purpose of this study was to explore whether an online brain health training, SMART, could promote mental well-being in two groups of adults, those with and those without mental illness. This study compared baseline characteristics between these groups as well as post-training changes in mental health and cognitive outcomes. Also investigated was whether greater cognitive gains in clarity predicted greater improvements in mental health outcomes.

### 4.1 Examining the need for mental health promotion for the general population

The first aim was to compare baseline mental health and cognition between groups. As expected, adults with mental illness reported significantly greater psychological distress and lower resilience, quality of life, meaningful activity, and cognitive clarity than those without mental illness. These results are consistent with prior studies showing poorer cognitive performance (Keefe et al., 2011; Qureshi et al., 2011; Rock et al., 2014; Zainal and Newman, 2018) and diminished well-being (Berghofer et al., 2020; Comer et al., 2011; Dong et al., 2019; IsHak et al., 2012; Prigent et al., 2014) among individuals with mental illness across the continuum of care. Although treatment status was unknown, the group with mental illness demonstrated overall lower performance on both well-being and cognitive measures. Notably, the group without mental illness also reported some psychological distress, about half the level of the clinical group (as shown in Figure 2), highlighting the value of proactive mental health promotion before symptoms reach a clinical threshold.

### 4.2 Impact of SMART brain training on mental health

The second aim was to examine post-training changes in mental health and cognition. SMART emerged as a promising mental health promotion tool for both groups, producing significant reductions in psychological distress and increases in resilience, quality of life, and meaningful activity. These gains were dose-dependent, with greater improvements linked to greater training utilization.

Importantly, baseline mental illness status did not affect outcomes, indicating similar benefits across those with and without mental illness despite initial differences in well-being and clarity. Unlike many prior studies that focus on study groups with a single diagnosis (e.g., Browning et al., 2011; McGurk et al., 2007; Wykes et al., 2011), this study highlights the value of brain training as a universal mental health promotion strategy deployable for those with and without mental illness, including those with comorbid mental illness diagnoses. This is particularly relevant given the high rates of comorbidity in the general population (30–50%; Kessler et al., 2005; Bagalman and Napili, 2014) and diagnostic instability across the life span (Caspi et al., 2020). These findings also extend emerging evidence that brain training can enhance well-being in non-clinical individuals (e.g., Wolinsky et al., 2009; Nakagami et al., 2024; Young et al., 2021; Laane et al., 2022).

Past explorations of the effects of cognitive training on well-being include only one or two components of mental health, with a focus on measuring negative symptoms (Nakagami et al., 2024; Ronold et al., 2022; Samuelson et al., 2021; Segrave et al., 2014; Wolinsky et al., 2009). This study took a more comprehensive approach by using multiple outcome measures for mental health, capturing both negative symptoms of mental illness (i.e., reduction in depressive, anxiety, and stress symptoms), but additionally, including measures of positive mental well-being (i.e., resiliency, quality of life, and engagement in meaningful activities). The finding that participants, regardless of group, demonstrated significant gains in all four measures of mental health, suggests that online SMART may be a particularly impactful tool for mental health promotion, with potential to enhance both mood and flourishing (through greater quality of life, resilience, and meaningful engagement).

Quality of life and engagement in meaningful activities are central to both prevention and treatment models of mental health. Quality of life is consistently reported as lower among individuals with mental illness compared to the general population (Berghofer et al., 2020; Comer et al., 2011; Dong et al., 2019; IsHak et al., 2012). Increasingly, practitioners call for quality of life to be considered a key outcome in patient-centered care, as improvements can occur even when complete remission from illness is not possible (Berghofer et al., 2020; Michalak et al., 2006; Purkaple et al., 2016). Similarly, engagement in meaningful activities has been identified as a protective factor: greater meaning in life is linked to lower depression and anxiety in both clinical and non-clinical populations (Boreham and Schutte, 2023) and is associated with better treatment adherence, reduced suicidal ideation, and higher quality of life for those with mental illness (Costanza et al., 2020; Tali et al., 2009). Resilience is also a critical component of mental health promotion. Resilience has been shown to protect against suicide risk in psychosis (Johnson et al., 2010) and in depression and anxiety disorders (Min et al., 2015), as well as in adolescents and young adults (Nrugham et al., 2010). More broadly, resilience may also act as a buffer against the onset of mental disorders and psychological distress (Arnetz et al., 2013; Hjemdal et al., 2006; Vazquez et al., 2024), supporting individuals’ ability to recover and adapt following challenges (Arnetz et al., 2013; Hjemdal et al., 2006; Loprinzi et al., 2011). By demonstrating improvements across symptom reduction and positive well-being indicators, this study highlights the potential of SMART as a multidimensional mental health promotion intervention.

### 4.3 Impact of SMART brain training on clarity

The second aim also explored change in clarity, a measure of high-level, functional cognitive performance. Results indicated differential responses to training between participants with and without mental illness. Among those without mental illness, individuals who completed the core SMART training (at least five sections) showed growth in clarity, suggesting that full exposure to the cognitive strategies may be necessary to drive improvements in higher-order cognitive performance. In contrast, participants with mental illness did not exhibit notable gains in clarity, even after completing the core training. This was unexpected, as it was hypothesized that individuals with mental illness would demonstrate greater improvements given their lower baseline scores and presumed greater capacity for change.

One possible explanation for the lack of clarity gains among participants with mental illness is that starting with lower baseline clarity may affect the acquisition or implementation of the SMART strategies. In general, prior research has shown mixed patterns in how baseline levels influence response to cognitive training. Some studies report a “compensation effect,” where individuals with lower baseline function exhibit greater gains, while others find a “magnification effect,” where those with higher baseline ability benefit more (Borella et al., 2017; Traut et al., 2021). The current findings suggest that online SMART may follow the magnification pattern: participants without mental illness, who entered with higher clarity, demonstrated greater improvements. Borella et al. (2017) reported a similar magnification effect for cognitively demanding tasks, where individuals with stronger baseline working memory showed greater improvements in executive function skills requiring complex information processing. In contrast, less demanding tasks (e.g., short-term recall) tended to show a compensatory effect, with greater growth among those starting lower. Given that clarity reflects complex, high-level cognition, these findings provide further support for a magnification effect in executive function training.

Consistent with this consideration, Vinogradov et al. (2012) hypothesized that basic cognitive functions must reach a certain threshold before individuals with impaired neural systems, such as those with mental illness, can derive generalizable benefits from top-down cognitive training. Future research should examine whether combining bottom-up cognitive training (targeting foundational functions such as attention or processing speed) with top-down strategy-based training (SMART) yields greater improvements in clarity among individuals with mental illness. Such an approach may help ensure that participants have the necessary cognitive foundation to fully benefit from higher-order strategy instruction.

It was unexpected that participants with mental illness did not show improvements in clarity, given that prior studies of SMART training among individuals with bipolar disorder (Venza et al., 2016) and post-traumatic stress disorder (Samuelson et al., 2020, 2021) reported positive gains in abstract reasoning and executive function. One possible explanation is that the in-person delivery format used in these previous protocols may have been more effective at stimulating change in high-level cognition due to the ability to tailor training in real time. In these studies, small group sessions allowed clinicians to adjust pacing, examples, and practice activities based on participant feedback, thereby aligning training demands with individual ability. This interpretation aligns with learning theories emphasizing that training is most effective when matched to a learner’s capacity (Vygotsky, 1978; Bjork and Bjork, 2011; Piaget, 1964; Sweller, 1988). Without real-time adjustment, the self-directed online SMART may have been less effective for participants with mental illness. Future iterations could integrate adaptive technologies to tailor pacing and feedback in response to participant performance.

Additionally, participants with mental illness engaged in less overall training compared to those without mental illness, which may have further contributed to the absence of observed gains. Lower training exposure could reflect barriers such as reduced motivation or difficulty sustaining attention in a self-directed format. The online, self-paced nature of SMART may also impose greater cognitive load, particularly for individuals with mental illness. In contrast, in-person delivery may reduce this cognitive burden by providing external structure, scaffolding, and immediate support. Without real-time adjustment and sufficient training dosage, the self-directed online SMART may have been less effective for participants with mental illness. Future iterations could integrate adaptive technologies (e.g., AI driven coaching) and structured supports (e.g., additional personalized notifications, gamified training elements). Alternatively, training courses could be adapted for certain populations – such as tailored pacing or including discrete, bottom-up cognitive training to complement the strategy training – to reduce cognitive load and enhance engagement in this population.

Another distinction is that prior in-person SMART studies used relatively homogeneous samples, focusing on participants with a single diagnosis (e.g., PTSD or TBI), while excluding individuals with comorbid psychotic, bipolar, or substance use disorders (Samuelson et al., 2020, 2021). In contrast, the present study included a diagnostically diverse sample, including participants with multiple comorbidities. It is possible that SMART may be more effective for improving abstract reasoning and problem solving in some diagnostic groups than others. Future research should therefore examine diagnostic subgroups to examine for whom online SMART is most effective in enhancing high-level cognitive function.

### 4.4 Relationship between cognitive function and mental health

Much remains to be explored regarding the relationship between cognitive and mental health outcomes. Findings from aim 3 aligned with the hypothesis that improvements in clarity would predict gains in psychological distress, resilience, quality of life, and engagement in meaningful activities; however, clarity explained only a small proportion of the variance (~2–5%). When examined by group, clarity gains predicted improvements in resilience and quality of life for both those with and without mental illness, but were uniquely associated with reduced psychological distress in the mental illness group and with greater engagement in meaningful activities in the non-mental illness group. These findings support Keshavan et al.’s (2014) model, which posits that cognitive training strengthens impaired neural systems, enhancing cognition and enabling greater community function. Specifically, results from the current study suggest that improving high-level cognition may bolster resilience and quality of life for those with mental illness, while also extending benefits to broader aspects of well-being for those without mental illness. Further research is needed to test and refine this model.

Several factors may explain why changes in cognitive function accounted for only a small proportion of the variance in mental health outcomes. Mental health is multifactorial, influenced not only by cognition but also by genetics, physical health, environmental stressors, social support, coping mechanisms, and engagement in interventions (Guintivano and Kaminsky, 2016; Mrazek and Haggerty, 1994; Kandasamy et al., 2020; Pevalin and Goldberg, 2003; Santarnecchi et al., 2018). These factors may contribute more strongly to improvements in well-being than cognitive gains alone. Future research should capture how supportive features of SMART—such as optional coaching sessions, daily habit tracking, and notification reminders that may simulate social support—interact with mental health outcomes. For example, SMART training encourages goal setting and equips participants with the executive function tools to work towards their goals – including a feature to track daily habit building. Therefore, the strategy-based training could act as a behavioral activation (BA) intervention which improves mood by increasing reward system activation and decreasing behavioral patterns of inactivity (Dimaggio and Shahar, 2017; Jacobson et al., 1996).

Measurement sensitivity may also play a role, as the clarity composite emphasizes higher-order reasoning and problem-solving, potentially missing improvements in specific cognitive processes (e.g., cognitive flexibility, selective attention) that more directly support mental health. Moreover, improvements in clarity may influence well-being indirectly, through downstream effects on other functions or behaviors, such as improvements in sleep impacting both mental health and cognition. Finally, because participants with mental illness did not show significant clarity gains, the regression model likely underestimates the true contribution of cognitive improvements to mental health in this group. Future research should examine these dynamics more closely and consider targeted adaptations to strengthen clarity outcomes in populations with mental illness.

### 4.5 Public health considerations

The findings from this study are encouraging and suggest that online brain health training may serve as an effective mental health promotion tool. However, additional research is needed to evaluate its effectiveness as a universal mental health promotion intervention, particularly given the limitations of the current sample. Participants were relatively homogeneous, consisting primarily of White (88.4%), female (81.6%), and highly educated individuals, with only 20.8% reporting less than a bachelor’s degree. As a result, the generalizability of these findings to more diverse and lower socioeconomic status (SES) populations is limited. These considerations are particularly important given that mental health is strongly shaped by social determinants including income, education, housing and food insecurity, social support, discrimination, and environmental exposures, which disproportionately affect marginalized and underrepresented populations (Kirkbride et al., 2024). Individuals experiencing these structural and contextual stressors may differ not only in mental health risk, but also in access to resources necessary for engagement in online interventions, such as time, stable internet access, and supportive environments. Therefore, future research should examine the effectiveness, accessibility, and cultural relevance of SMART training among more diverse populations and among individuals experiencing elevated social and environmental risk factors.

In addition to structural factors, participant-level characteristics may also influence engagement and outcomes. Further research is needed to examine the role of self-agency (i.e., belief in one’s ability to effect change) and motivation (e.g., personal interest or curiosity) as both potential confounding variables as well as potential outcomes of training. Individuals with higher baseline self-agency and motivation may have been more likely to enroll in the study and engage consistently with the intervention. At the same time, the intervention itself is intentionally designed to strengthen both constructs. Participants in the present sample likely demonstrated relatively high baseline motivation, as evidenced by completion of the 6-month training protocol and post-assessment. However, the platform also includes features intended to promote sustained engagement and motivation, including coaching sessions, daily habit tracking, goal setting, and notification nudges. Supporting this interpretation, Cook et al. (2026) found in a large longitudinal sample of Brain Health Project users that participants with low initial utilization increased their engagement over time. Similarly, individuals with greater self-agency may be overrepresented in the sample, given that they may be more likely to initiate and adhere to self-directed interventions. Nevertheless, SMART strategy training incorporates components specifically designed to enhance self-agency. For example, abstraction strategies such as “zooming out” may increase perceived control and empowerment, consistent with evidence that abstract thinking enhances one’s sense of power (Smith et al., 2008). Future research should include repeated measures of self-agency and motivation to better disentangle their roles as predictors, mechanisms, and outcomes of intervention engagement and to clarify the appropriateness of SMART for individuals with differing characteristic profiles.

Finally, additional research is needed to better understand the broader public health impact of online SMART training. The small-to-medium effect sizes observed in this study are consistent with those reported in other public mental health promotion interventions (Sodi et al., 2025). Importantly, even small effect sizes may yield meaningful population-level benefits when interventions are scalable and broadly accessible (Carey et al., 2023). However, the extent to which these findings translate into measurable improvements in public mental health remains unclear. Future collaborations with large public health systems and community organizations could help evaluate the real-world reach, scalability, and long-term impact of online brain health training.

### 4.6 Limitations and future directions

Several limitations should be considered. First, the study relied on participant self-report for mental illness and did not collect information on past or ongoing treatments, medications, or clinical assessments. This lack of verified information may have led to under- or over-reporting and precluded accounting for the influence of concurrent treatments. The sample was also relatively homogeneous, limiting the generalizability of the findings to more diverse and lower-SES populations. Additionally, this study included community-dwelling individuals with generally low levels of psychological distress, limiting generalizability to more severe or inpatient populations. The study examined only immediate impacts of the intervention, without assessing maintenance of effects over time, and did not evaluate how training features – such as notification use, coaching, resource access, or pacing – may influence outcomes. Data for this study was limited to participants who completed at least two assessments; therefore, we do not know causes of attrition or characteristics of participants likely to be unsuccessful with this form of online brain training. Similarly, there is a need to explore the role of motivation and self-efficacy in training participation and utilization. Additionally, the cognitive measure used (clarity) is a composite of high-level functional performance; the contribution of individual components was not examined, and nuanced improvements in specific cognitive functions may not have been captured. Finally, as an observational quasi-experimental study, this design limits the ability to establish causal relationships. Future research using randomized controlled trials is needed to confirm these findings.

Future research on online SMART should explore ways to enhance its impact on cognition for individuals with mental illness, including combining it with bottom-up cognitive training or adapting the program to be more flexible, for example using AI to tailor pacing and feedback to individual progress. Investigating effects within specific diagnostic groups is also important, as distinct disorders may respond differently to top-down training or require supplemental interventions to improve clarity, such as exploring whether human-led coaching may be more appropriate for those with certain pre-existing mental health conditions. Given the multifactorial nature of mental health, future studies should measure environmental stressors, social support, and concurrent treatments, and examine how different components of clarity relate to changes in well-being. Finally, research should investigate the interplay between mental health domains, as improvements in resilience, quality of life, or well-being may mutually reinforce each other (Chuang et al., 2023; Wood and Joseph, 2010). Using multiple measures and longitudinal designs will help identify the most effective strategies for promoting lasting mental health benefits.

## 5 Conclusion

This study examined the mental health promotion effects on online brain health training for individuals with and without mental illness, demonstrating that a single promotion intervention may benefit the public regardless of baseline mental health. Findings support the consideration of SMART, a top-down executive function intervention, as a universal tool to target improvements in symptoms of psychological distress, resilience, quality of life, and engagement in meaningful activities. To our knowledge, this is the first study to assess top-down cognitive training across both populations, showing benefits across the adult age range. These results suggest that strategy-based cognitive interventions can play a key role in public mental health promotion. Future research should refine virtual training to better support individual needs, particularly for enhancing cognitive function among those with mental illness, advancing toward precision brain health. Scalable, effective brain health interventions may offer a proactive approach to mitigating the growing mental health crisis.

## Data Availability

The raw data supporting the conclusions of this article will be made available by the authors, without undue reservation.
