# Supplementary material for: Improving mental health outcomes through online brain health training in adults with or without mental illness
Source: Front Psychol. 2026 Jun 4;17:1826717. doi: 10.3389/fpsyg.2026.1826717 (PMC13277663; doi:10.3389/fpsyg.2026.1826717)
Supplement: Supplementary file 1 [file Data_Sheet_1.PDF]

Supplementary Materials

Supplementary Figure S1.

Summary of Balance for All Data:

	Means Treated	Means Control	Std. Mean Diff.	Var. Ratio	eCDF Mean	eCDF Max
distance	0.2595	0.2318	0.3674	1.2429	0.0898	0.1752
age_group[18,36.6]	0.0757	0.0440	0.1198	.	0.0317	0.0317
age_group(36.6,55.2]	0.1730	0.1760	-0.0079	.	0.0030	0.0030
age_group(55.2,73.7]	0.6162	0.5922	0.0494	.	0.0240	0.0240
age_group(73.7,92.3]	0.1351	0.1878	-0.1541	.	0.0527	0.0527
education_groupLess than Bachelor	0.2054	0.1269	0.1943	.	0.0785	0.0785
education_groupBachelor's degree	0.2865	0.3976	-0.2458	.	0.1111	0.1111
education_groupGreater than Bachelor	0.5081	0.4755	0.0653	.	0.0326	0.0326
gender_numeric	0.1784	0.2623	-0.2191	.	0.0839	0.0839

Summary of Balance for Matched Data:

	Means Treated	Means Control	Std. Mean Diff.	Var. Ratio	eCDF Mean	eCDF Max	Std. Pair Dist.
distance	0.2595	0.2580	0.0198	1.0991	0.0014	0.0162	0.0198
age_group[18,36.6]	0.0757	0.0703	0.0204	.	0.0054	0.0054	0.0204
age_group(36.6,55.2]	0.1730	0.1784	-0.0143	.	0.0054	0.0054	0.0143
age_group(55.2,73.7]	0.6162	0.6162	0.0000	.	0.0000	0.0000	0.0000
age_group(73.7,92.3]	0.1351	0.1351	0.0000	.	0.0000	0.0000	0.0000
education_groupLess than Bachelor	0.2054	0.2108	-0.0134	.	0.0054	0.0054	0.0669
education_groupBachelor's degree	0.2865	0.2865	0.0000	.	0.0000	0.0000	0.0000
education_groupGreater than Bachelor	0.5081	0.5027	0.0108	.	0.0054	0.0054	0.0541
gender_numeric	0.1784	0.1892	-0.0282	.	0.0108	0.0108	0.0282

Sample Sizes:

	Control	Treated
All	591	185
Matched	185	185
Unmatched	406	0
Discarded	0	0

Figure S1. Reduced mean difference shows the effectiveness of matching method. *Note.* "Treated" refers to the group with mental illness while "Control" refers to the group without mental illness. The matching procedure effectively reduced mean differences across covariates to below 0.03.

Supplementary Figure S2.

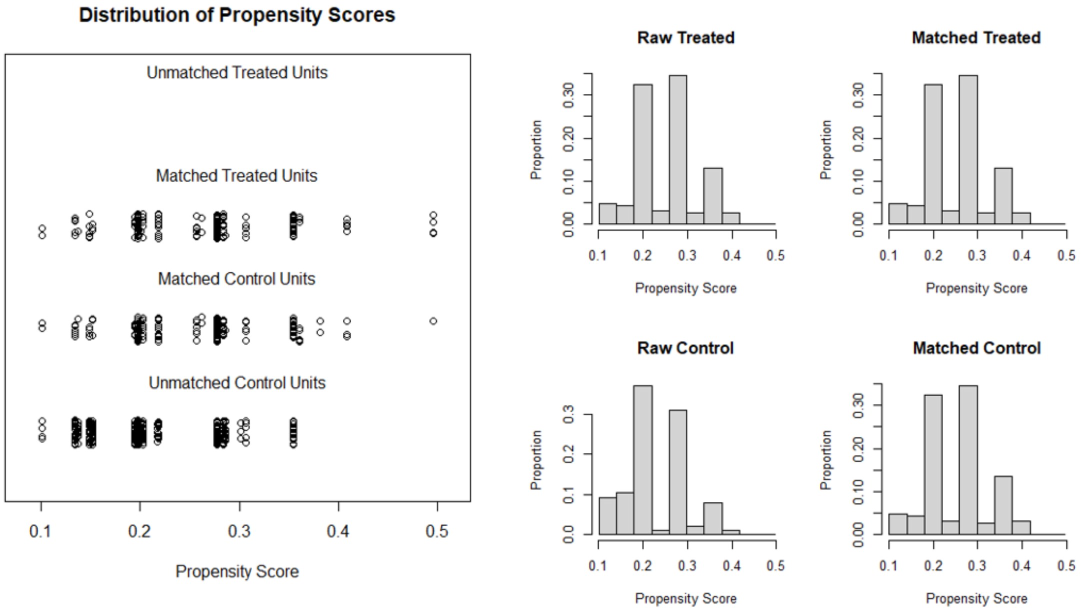

Figure S2. Visual representation of matching procedure. *Note.* The jitter plot (left) and histograms (right) indicate comparable propensity score balance between participants with and without mental illness. The "Treated" group includes individuals with mental illness, whereas the "Control" group includes those without mental illness. "Raw" refers to the full, unmatched sample, and "Matched" refers to the subset of participants retained following the matching procedure.

Supplementary Figure S3.

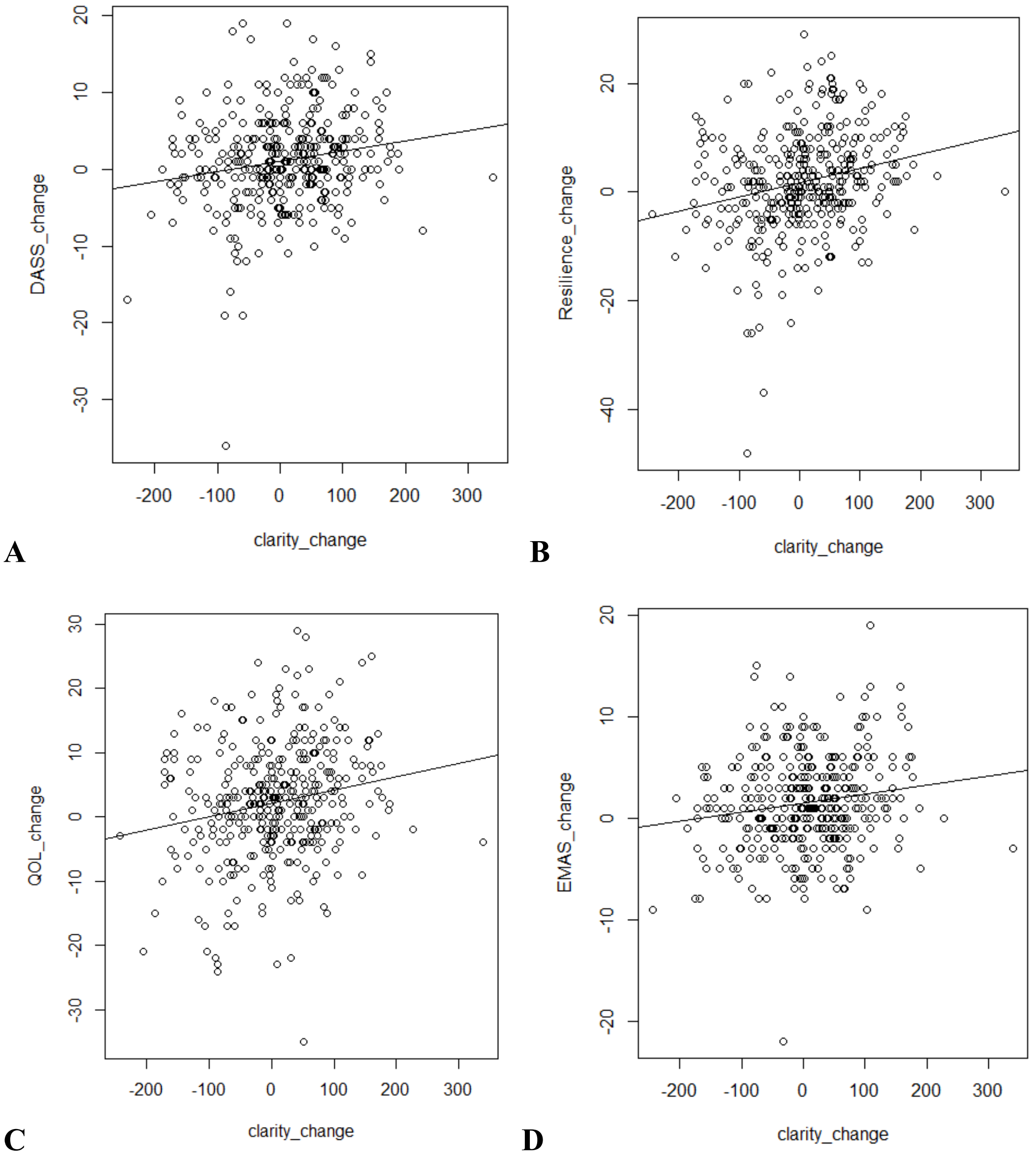

Figure S3. Aim 3 regressions between clarity change and mental health variables. *Note.* A) Correlation of clarity change and symptom (DASS-21) change; DASS_change is time 1 subtracted from time 2 results, therefore higher values indicate more reduction in symptoms. B) Correlation between clarity change and resilience change. (C) Correlation between clarity change and quality of life (QOL) change. (D) Correlation between clarity change and engagement in meaningful activities (EMAS) change.

Supplementary Table S1

Interaction Effects of Demographics on Change in Mental Health Outcomes and Clarity

Outcome	Demographic Predictor	Estimate (β)	SE	t(df)	p
Symptoms of psychological distress	Age x Time	0.03	0.02	1.34(364)	.180
	Gender x Time	0.61	0.83	0.74(364)	.461
	Time x Education: (GB vs. B)	-0.89	0.75	-1.20(364)	.232
	Time x Education (LB vs. B)	-0.99	0.92	-1.08(364)	.280
	Time x Education (GB vs. LB)	0.10	0.84	0.12(364)	.906
Resilience	Age x Time	-0.12	0.04	-3.25(364)	.001*
	Gender x Time	0.83	1.26	0.66(364)	.512
	Time x Education: (GB vs. B)	2.54	1.14	2.23(364)	.026*
	Time x Education (LB vs. B)	0.70	1.40	0.50(364)	.617
	Time x Education (GB vs. LB)	1.84	1.28	1.44(364)	.152
Quality of Life	Age x Time	-0.01	0.04	-0.28(364)	.779
	Gender x Time	0.42	1.23	0.34(364)	.735
	Time x Education: (GB vs. B)	0.57	1.11	0.51(364)	.608
	Time x Education (LB vs. B)	0.06	1.37	0.04(364)	.965
	Time x Education (GB vs. LB)	0.51	1.25	0.41(364)	.683
Engagement in Meaningful Activities	Age x Time	-0.01	0.02	-0.61(364)	.542
	Gender x Time	0.24	0.63	0.38(364)	.706
	Time x Education: (GB vs. B)	0.66	0.57	1.16(364)	.245
	Time x Education (LB vs. B)	0.91	0.70	1.30(364)	.193
	Time x Education (GB vs. LB)	-0.25	0.64	-0.39(364)	.696
Clarity	Age x Time	-0.72	0.32	-2.24(364)	.026*
	Gender x Time	-7.33	11.14	-0.66(364)	.511
	Time x Education: (GB vs. B)	6.63	10.05	0.66(364)	.510
	Time x Education (LB vs. B)	-7.42	12.36	-0.60(364)	.548
	Time x Education (GB vs. LB)	14.06	11.29	1.24(364)	.214

Note. Values represent fixed-effect interaction terms from linear mixed-effects models examining moderation of change from baseline to post-test. GB = Greater than Bachelor's degree, B = Bachelor's degree, LB = Less than Bachelor's degree. *Statistically significant for p < .05.

Supplementary Table S2. Linear Mixed-Effects Full Model Results for Clarity Among Core-Trained Participants

	Estimate Value (Std. Error)	*t*-value (DF)	*p* value
Time	9.83 (8.35)	1.18 (171)	.240
Effect of time without mental illness only	18.41 (9.99)	1.84 (171)	.067~
Effect of time with mental illness only	1.25 (10.58)	0.12 (171)	.906
Time x Mental illness	-17.15 (12.05)	-1.42 (171)	.156
Training	-7.44 (9.74)	-0.76 (169)	.446
Mental illness x Training	-31.84 (19.59)	-1.63 (169)	.106
Age x Time	-0.77 (0.46)	-1.67 (171)	.096~
Gender x Time	-15.83 (16.21)	-0.98 (171)	.330
Education: Greater than Bachelor's – Bachelor's degree x Time	0.26 (14.00)	0.02 (171)	.985
Education: Less than Bachelor's – Bachelor's degree x Time	-25.94 (17.58)	-1.48 (171)	.142
Education: Greater than Bachelor's – Less than a Bachelor's degree x Time	26.19 (16.09)	1.63 (171)	.105

Note. *Statistically significant for $p < .05$, ~Marginal at $p <.05$
